# Prognostic Impact of POLE Exonuclease-Domain Mutations in Endometrial Cancer: A Systematic Review and Meta-Analysis

**DOI:** 10.3390/cancers18040597

**Published:** 2026-02-11

**Authors:** Ioana Hurmuz, Robert Barna, Aura Jurescu, Bianca Natarâș, Dorela-Codruța Lăzureanu, Iuliana-Anamaria Trăilă, Alexandru-Marius Furău, Sorina Tăban, Alis Dema

**Affiliations:** 1 Department of Microscopic Morphology-Anatomic Pathology, ANAPATMOL Research Center, “Victor Babes” University of Medicine and Pharmacy, 300041 Timisoara, Romania; ioana.hurmuz@umft.ro (I.H.); robert.barna@umft.ro (R.B.); jurescu.aura@umft.ro (A.J.); bianca.nataras@umft.ro (B.N.); lazureanu.dorela@umft.ro (D.-C.L.); taban.sorina@umft.ro (S.T.); dema.alis@umft.ro (A.D.); 2 Department of Pathology, “Pius Brînzeu” County Clinical Emergency Hospital, 300723 Timisoara, Romania; 3 Medical Oncology Department, Emergency Clinical County Hospital of Arad, 310029 Arad, Romania; furau.marius@uvvg.ro; 4 Department of Oncology, “Vasile Goldis” Western University of Arad, 310414 Arad, Romania

**Keywords:** POLE mutation, endometrial cancer, exonuclease domain, ultramutated subtype, molecular classification, prognosis, survival outcomes

## Abstract

**Background/Objectives**: Traditional histopathological classification of endometrial cancer (EC) exhibits limited prognostic precision due to interobserver variability and incomplete reflection of tumor biology. The Cancer Genome Atlas (TCGA) introduced molecular subtypes, with POLE-ultramutated tumors showing superior outcomes. This systematic review and meta-analysis assesses the prognostic impact of pathogenic/likely pathogenic POLE exonuclease-domain mutations (EDM) on survival in EC. **Methods**: PRISMA 2020-compliant search of PubMed, Embase, and Web of Science (2015–2025) identified 20 studies (*n* = 7708 EC patients; 159 POLE-mutant, 2.1%). Eligibility: Adult EC patients with POLE EDM vs. non-POLE, reporting OS/PFS/DFS/RFS/CSS hazard ratios (HR). ROBINS-I assessed bias; random-effects meta-analysis pooled multivariable HR. **Results**: The meta-analysis revealed a significantly reduced risk of death in POLE-mutant endometrial cancer patients, with an OS pooled hazard ratio (HR) of 0.35 (95% CI 0.21–0.58; I^2^ = 17.9%) across eight studies. Disease control endpoints (DFS/PFS/RFS) from 10 studies showed an even more substantial benefit, with a pooled HR of 0.22 (95% CI 0.12–0.41; I^2^ = 0%). Cancer-specific survival HRs ranged from 0.00 to 0.32 across four studies, often with zero events in POLE cohorts. ROBINS-I bias was low to moderate; heterogeneity stemmed from the comparators and stages. **Conclusions**: POLE-EDM confers a robust, favorable prognosis across EC stages, supporting molecular risk stratification and treatment de-escalation.

## Simple Summary

Endometrial cancer, a common gynecological malignancy, has traditionally been classified by its appearance under the microscope. Still, this approach often misses key biological details that predict disease progression. Specific mutations in the POLE gene, which helps proofread DNA during cell division, create highly unstable tumors, yet surprisingly lead to better patient outcomes. This study reviews existing research and combines data from multiple studies to clarify the actual survival benefits of these patients with POLE mutations. The findings could help doctors better classify patients based on risk level, potentially allowing safer reductions in aggressive treatments for those with favorable mutations, improving care, and guiding future clinical trials.

## 1. Introduction

Traditional histopathological classification of endometrial carcinoma (EC) (histotype, grade, and FIGO stage) shows limited reproducibility and prognostic precision, primarily due to interobserver variability and insufficient reflection of tumor biology, reducing its value for risk stratification and adjuvant treatment decisions [1,2]. The Cancer Genome Atlas (TCGA) introduced a four-group molecular classification: POLE-ultramutated, MMR-deficient (MMRd), copy-number low/no specific molecular profile (NSMP), and p53-abnormal. POLE-mutant tumors show excellent outcomes and p53-abnormal tumors the poorest prognosis, while MMRd and NSMP represent intermediate-risk groups [3,4,5]. Also, EC exhibits a diverse spectrum of somatic mutations, with key driver genes playing pivotal roles in tumorigenesis and progression. Among the most frequently mutated genes are PTEN (approximately 57%), PIK3CA (51%), TP53 (30%), KRAS (23%), CTNNB1 (21%), FGFR2 (13%), FBXW7 (10%), and RB1 (9%). These mutations contribute to EC heterogeneity and have implications for prognosis and therapeutic strategies [6]. Clinical implementation has been enabled by surrogate classifiers such as the Proactive Molecular Risk Classifier for Endometrial Cancer (ProMisE) and TransPORTEC, which combine immunohistochemistry for MMR proteins and p53 with targeted POLE sequencing, enabling routine molecular classification in diagnostic pathology [3,5,7].

Clinically, POLE-mutated ECs are often associated with high-grade endometrioid histology, early-stage presentation, and a favorable prognosis. Notably, data from the PORTEC-3 trial indicate a 5-year recurrence-free survival (RFS) rate of 98% for patients with POLE-mutated ECs [5,8]. POLE-mutant tumors are characterized by a hypermutated and highly immunogenic phenotype, and emerging evidence, including case reports, small clinical series, and biomarker-focused reviews, suggests that POLE mutations may be associated with durable responses to anti-PD-1 therapy in appropriately selected clinical contexts [9,10].

Accordingly, international guidelines (ESGO, ESTRO, ESP, and ESMO) now recommend molecular classification for all ECs, and the 2023 FIGO staging system formally incorporates molecular features, underscoring their established prognostic and therapeutic relevance [3,11,12,13].

DNA polymerase epsilon (POLE) exonuclease-domain mutations (EDMs) in EC are associated with a favorable prognosis and are driven by distinct biological mechanisms, occurring in approximately 5–15% of EC cases [14,15]. Pathogenic variants affecting the proofreading domain (P286R, V411L) disrupt replication fidelity, resulting in an ultramutated phenotype (>100 mutations/Mb) characterized predominantly by single-nucleotide variants and a unique mutational signature enriched in C>A and T>G transversions. Unlike mismatch repair-deficient tumors, POLE-mutant cancers are typically microsatellite-stable and exhibit the highest tumor mutational burden among molecular subtypes, reflecting a fundamentally different genomic instability process [16,17,18,19,20].

The ultramutated genotype generates a high neoantigen load, leading to a highly immunogenic tumor microenvironment with dense CD8+ T-cell infiltration, increased tumor-infiltrating lymphocytes (TILs), and upregulation of cytotoxic effector molecules and immune checkpoint pathways. This enhanced immunogenicity promotes effective immune surveillance and durable tumor control, providing a mechanistic explanation for the exceptionally low recurrence rates and disease-specific mortality observed in POLE-mutant EC, independent of conventional clinicopathologic risk factors. Consistently, systematic reviews and large cohort studies demonstrate superior overall, progression-free, and disease-specific survival compared with other molecular subtypes, supporting current recommendations for treatment de-escalation in early-stage POLE-mutant disease [7,21,22,23,24].

The prognostic impact of the POLE mutation in EC remains unclear due to several methodological limitations in the literature. First, outcome reporting is highly heterogeneous, with studies variably assessing overall survival (OS), disease-free survival (DFS), progression-free survival (PFS), and cancer-specific survival (CSS) and using inconsistent effect measures, including hazard ratios (HR), log-rank *p*-values, or Kaplan-Meier curves without quantitative estimates. Studies differ in their use of multivariable-adjusted versus unadjusted analyses, and adjustment strategies are often incompletely reported, limiting comparability across cohorts and precluding robust meta-analytic synthesis, particularly across disease stages and risk groups [8,23,24,25,26]. Many cohorts report zero recurrences or cancer-related deaths among POLE mutation cases, especially in early-stage and high-grade disease, resulting in undefined or unstable HR and wide CI [8,24]. Although statistical approaches such as continuity corrections or exclusion of zero-event studies have been applied, these methods may introduce bias and are inconsistently implemented or discussed [23,25]. Comparator groups vary substantially across studies: some analyses compare POLE-mutation tumors only to NSMP cases, others compare POLE-mutation tumors only to pooled non-POLE-mutation tumors, and still others selectively include or exclude MMRd and p53-abnormal subtypes, despite their markedly different prognoses [23,27]. This variability directly affects effect estimates and clinical interpretation. Collectively, the lack of standardized outcome definitions, inconsistent comparator selection relative to ESGO/ESTRO/ESP recommendations, and inadequate handling of zero-event data underpin persistent uncertainty and justify further systematic review and methodologically rigorous meta-analysis across disease settings [3,8,25,26,27].

The primary objective of this study was to systematically review and quantitatively synthesize the available evidence on the prognostic impact of pathogenic and likely pathogenic POLE EDM in EC. Specifically, this systematic review and meta-analysis aimed to evaluate the association between POLE-mutant status and key survival outcomes, including OS, PFS, or DFS, and CSS, across diverse clinical settings and disease stages. In addition, this study sought to critically examine methodological sources of heterogeneity in the existing literature (variability in outcome reporting, the presence of zero-event POLE-mutant cohorts, and inconsistencies in comparator group definitions) that may influence effect estimates and clinical interpretation. By integrating data from observational cohorts and real-world studies using a rigorous, PRISMA-compliant framework, this work aims to provide a robust, clinically meaningful synthesis of current evidence to inform molecular risk stratification, guide therapeutic decision-making, and support future treatment de-escalation strategies in POLE-mutant EC.

Compared with prior systematic reviews and meta-analyses, this study provides a more clinically focused synthesis by restricting the analysis to pathogenic and likely pathogenic POLE EDMs, integrating multiple prespecified survival endpoints (OS, DFS/PFS/RFS, and CSS), explicitly addressing key methodological challenges in observational evidence and contextualizing the findings within the contemporary FIGO 2023 staging system and ESGO/ESTRO/ESP molecular risk stratification framework.

## 2. Materials and Methods

### 2.1. Protocol and Reporting Standards

This systematic review and meta-analysis were conducted and reported in accordance with the Preferred Reporting Items for Systematic Reviews and Meta-Analyses (PRISMA) 2020 guidelines [28]. The review protocol is registered in the International Prospective Register of Systematic Reviews (PROSPERO) under registration number CRD420251273994 and is available at https://www.crd.york.ac.uk/PROSPERO/view/CRD420251273994, accessed on 31 January 2026. This study was designed to systematically evaluate the prognostic impact of pathogenic and likely pathogenic POLE EDM on survival outcomes in patients with EC.

### 2.2. Eligibility Criteria

The eligibility criteria were defined a priori according to the PICOS framework (population, intervention, comparator, outcomes, and study design).

Population: We included studies enrolling adult patients (≥18 years) with histologically confirmed EC, irrespective of tumor stage, histological subtype, grade, or treatment setting (early-stage, high-risk, advanced, or recurrent disease).

Exposure: The presence of pathogenic or likely pathogenic POLE EDM, as defined by the original studies using validated molecular testing methods. Studies evaluating POLE mutations outside the exonuclease domain or reporting variants of uncertain significance without precise pathogenic classification were excluded.

Comparator: EC without POLE EDM, defined either as pooled non-POLE tumors or as specific molecular subgroups NSMP, MMRd, or p35abn, as reported by the individual studies.

Outcomes: Studies that report at least one time-to-event survival outcome, such as OS, PFS, DFS, RFS, or CSS, with outcomes stratified by POLE mutation status or data that can be used to stratify by POLE mutation status.

It included original observational studies (prospective or retrospective cohort studies) and analyses of randomized clinical trial (RCT) cohorts that reported prognostic outcomes according to POLE mutation status. Studies were excluded if they did not represent original clinical research. Preclinical studies (in vitro or in vivo) and investigations not involving human EC were also excluded. Additionally, studies were excluded if they lacked sufficient molecular detail to allow reliable classification of POLE EDM status, evaluated non-pathogenic or non-exonuclease-domain POLE variants only, or did not provide data enabling meaningful prognostic interpretation within the scope of this review. Duplicate publications, overlapping cohorts with insufficiently distinct datasets, languages other than English, and studies with irretrievable full texts were excluded to avoid data redundancy and ensure methodological rigor (Table S1 in Supplementary Materials).

### 2.3. Information Sources and Search Strategy

A comprehensive and systematic literature search was conducted in the following electronic databases, PubMed (MEDLINE), Embase, and Web of Science, from 2015 to 2025. The search strategy was developed a priori and combined controlled vocabulary terms with free-text keywords related to POLE mutations, EC, and prognostic outcomes. Search terms included variations and synonyms of “POLE”, “DNA polymerase epsilon”, “exonuclease domain”, “endometrial carcinoma”, and survival-related outcomes such as “overall survival”, “progression-free survival”, and “prognosis”. Boolean operators (“AND”, “OR”) were used to combine concepts appropriately. The full search strategies for all databases are provided in the Supplementary Materials to ensure reproducibility (Table S2 in Supplementary Materials).

No restrictions were applied regarding publication year or geographic location. Only articles published in the English language were considered eligible. In addition to the reference lists of all included studies, two authors manually screened relevant review articles to identify additional eligible studies not captured by the electronic search.

### 2.4. Study Selection

**Figure 1:**
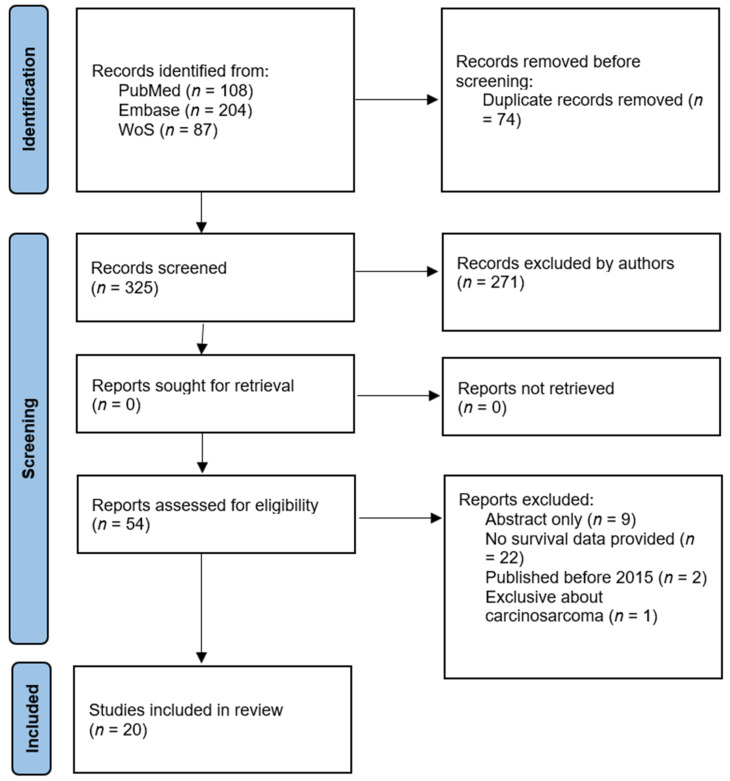
PRISMA flow diagram.

All records identified through the electronic database searches were imported into a reference management system, and duplicate records were removed before screening. Study selection was performed in a two-stage process by two authors. Any discrepancies at either stage were resolved through discussion and consensus. When necessary, a third senior author was consulted to reach a final decision. First, titles and abstracts were independently screened for eligibility using the predefined inclusion and exclusion criteria. The overall study selection process is summarized using a PRISMA 2020 flow diagram (Figure 1).

### 2.5. Data Extraction

For each included study, the following information was extracted: first author and year of publication; country and study design; inclusion period; total sample size and number of patients with POLE EDM; patient demographics; tumor stage, histology, and grade distributions; molecular classification subgroups; details of POLE mutation testing (genomic regions assessed, mutation definition, and sequencing methodology); and follow-up duration. Prognostic outcomes of interest, including OS, PFS, DFS, RFS, and CSS, were extracted when reported, along with the corresponding effect estimates (HRs with 95% confidence intervals (CIs)) and the variables included in multivariable adjustment models.

When multiple effect estimates were available, multivariable-adjusted HRs were preferentially extracted. If adjusted estimates were not reported, unadjusted estimates were collected and clearly identified. When quantitative effect estimates were unavailable or could not be reliably extracted, studies were retained for qualitative synthesis only.

### 2.6. Risk of Bias Assessment

The risk of bias of the included studies was assessed using the Risk of Bias in Non-randomized Studies of Interventions (ROBINS-I) tool, which is recommended for evaluating methodological quality in non-randomized studies reporting prognostic outcomes. The ROBINS-I tool evaluates bias across seven domains: confounding, participant selection, exposure classification, deviations from intended exposures, missing data, outcome measurement, and selection of the reported result. Risk-of-bias assessment was performed independently by two reviewers. Each domain was rated as low, moderate, high, or critical risk of bias, and an overall risk-of-bias judgment was assigned for each study according to ROBINS-I guidance. Any disagreements between reviewers were resolved through discussion and consensus.

The results of the risk-of-bias assessment are summarized in a table and presented in the Section 3 and in Supplementary Materials (Table S3). The risk-of-bias judgments were considered in the interpretation of the findings, particularly with respect to study design, sample size, event rates, and the robustness of the reported effect estimates.

### 2.7. Effect Measures and Statistical Analysis

The primary effect measure for quantitative synthesis was the HR with corresponding 95% CIs for time-to-event outcomes. When available, multivariable-adjusted HRs were preferentially extracted to minimize confounding. If adjusted estimates were not reported, unadjusted HRs were extracted and clearly identified. Studies that did not report extractable HRs with 95% CIs were included in the qualitative synthesis only. Meta-analyses were performed using a random-effects model, acknowledging the anticipated clinical and methodological heterogeneity across studies, including differences in patient populations, disease stage, molecular testing strategies, and comparator definitions. Separate meta-analyses were planned for OS and PFS/DFS/RFS, provided that a sufficient number of studies reported comparable effect estimates.

Statistical heterogeneity was assessed using Cochran’s Q test and quantified using the I^2^ statistic; values of approximately 25%, 50%, and 75% were interpreted as low, moderate, and high heterogeneity, respectively. Due to the limited number of studies and the rarity of POLE-mutant events, formal assessment of publication bias (e.g., funnel plots or Egger’s test) was not planned.

Comparator definitions varied across studies, including pooled non-POLE tumors, NSMP-only cohorts, and broader molecular subgroup comparisons. Given the rarity of POLE-mutant cases and the limited number of studies reporting stratified HRs by molecular subgroup, formal subgroup meta-analyses by comparator type were not feasible without substantially reducing statistical power. Therefore, a random-effects model was used to account for both clinical and methodological heterogeneity.

Studies reporting zero events in the POLE-mutant group were not included in pooled HR meta-analyses because effect estimates were not assessable or statistically unstable; however, these studies were retained for narrative synthesis to provide clinically relevant context for prognosis.

### 2.8. Synthesis Methods and Analytical Tools

All statistical analyses were conducted using Python (version 3.12). Meta-analytic computations and data handling were performed using the following libraries: pandas, numpy, scipy, statsmodels for meta-analytic models and variance estimation and matplotlib for graphical visualization of forest plots.

HRs were logarithmically transformed before pooling, and standard errors were derived from reported CIs using established methods. Pooled effect estimates and 95% CIs were calculated using inverse-variance weighting under a random-effects framework. Forest plots were generated to visually summarize individual study estimates and pooled results. Planned subgroup or sensitivity analyses stratified by comparator type were not performed due to the limited number of studies within each comparator category and the small number of POLE-mutant events, which would have resulted in unstable or non-informative pooled estimates. All analyses were performed using reproducible scripts, and statistical significance was defined as a two-sided *p*-value < 0.05.

## 3. Results

### 3.1. Study Selection and Overview of Incidence Evidence

The study selection process followed the PRISMA 2020 guidelines and is illustrated in Figure 1. The database search yielded 399 records (PubMed: *n* = 108; Embase: *n* = 204; Web of Science: *n* = 87). After removing 74 duplicates, 325 records were screened by title and abstract, resulting in the exclusion of 271.

A total of 54 full-text articles were assessed for eligibility; 34 were excluded due to a lack of relevant survival data, abstract-only reporting, publication before 2015, or an exclusive focus on uterine carcinosarcoma. Ultimately, 20 studies met the inclusion criteria and were included in the qualitative synthesis. Studies reporting extractable HR were considered eligible for quantitative synthesis, while those with non-estimable effect measures were retained for narrative synthesis only.

### 3.2. Characteristics of Included Studies

The 20 included studies were published between 2015 and 2025 and comprise a total of 7708 patients with EC, of whom 159 (2.1%) harbored pathogenic or likely pathogenic POLE EDM. Most studies were conducted in single countries, predominantly the United States (*n* = 5) [29,30,31,32,33], China (*n* = 3) [34,35,36] and Korea (*n* = 2) [37,38], followed by European countries including the Netherlands (*n* = 2) [39,40], and individual studies from Turkey [41], Canada [42], Brazil [43], Denmark [44], Norway [24], and Finland [45], as well as multinational or multicenter cohorts spanning Europe, North America, and Australia [5,46] (Table 1 and extended extracted data in Table S4 in Supplementary Materials).

Regarding study design, the majority were retrospective cohort studies (*n* = 17), with additional designs including retrospective analyses of RCT cohorts (*n* = 2) [34,35] and one prospective cohort study [38]. Inclusion periods varied across studies, generally spanning 5 to 15 years, reflecting heterogeneous accrual timelines. Sample sizes per study ranged from 72 to 1139, and the number of POLE-mutant cases per study ranged from 7 to 51. When reported, median or mean patient age at diagnosis was mainly in the sixth to seventh decade of life, consistent with the epidemiology of EC.

Most studies included primarily patients with early-stage disease, although several cohorts also included high-risk or advanced-stage cases. Histologically, the populations are dominated by EC, with variable inclusion of non-endometrioid subtypes such as serous or mixed-histology. Tumor grade distributions were heterogeneous, with multiple studies reporting a substantial proportion of high-grade tumors within the POLE-mutant subgroup.

All included studies report molecular classification data sufficient to identify POLE EDM, most commonly within the canonical exonuclease domain hotspots. Molecular testing was predominantly performed using NGS-based panels, although sequencing platforms, genomic coverage, and variant annotation criteria varied across studies. Definitions of pathogenic or likely pathogenic POLE mutations were based on study-specific criteria, typically incorporating established hotspot variants and functional annotation.

At the time of reporting, the median follow-up duration ranged from 23 to 131 months, allowing assessment of long-term survival outcomes. OS, DFS, PFS, RFS, and CSS were variably reported across studies, with several cohorts providing multiple survival endpoints.

Treatment modalities (surgery, adjuvant radiotherapy, chemotherapy, or combined chemoradiation) were reported heterogeneously across studies and were rarely stratified by POLE mutation status or potential multiple-classifier phenotypes, thereby limiting the comparability of treatment effects.

### 3.3. Molecular Features of POLEmut Cohorts

The total of 20 studies included molecular data, allowing classification of POLE EDM. Across these studies, the number of POLE-mutant cases per cohort ranged from 7 to 51, with most reporting 10–40 POLE-mutant tumors (Table 2). No included study reported tumors with concurrent molecular classifiers (e.g., POLE-mutant with simultaneous MMR deficiency or p53 abnormality); when addressed, such cases were typically excluded during molecular class assignment to preserve mutually exclusive TCGA-aligned categories, suggesting that either these phenotypes were absent or, more likely, not systematically reported across cohorts.

#### 3.3.1. Molecular Classification Frameworks

Molecular subgroup reporting was heterogeneous. TCGA- or ProMisE-aligned molecular classification was explicitly reported in 12 studies, including complete stratification into POLEmut, MMRd, p53-abnormal, and NSMP subgroups [5,31,32,33,36,37,38,40,41,43,45,46] (e.g., Stelloo et al., 2016; Leon-Castillo et al., 2020; Bosse et al., 2018; Han et al., 2024). Partial molecular classification—typically reporting POLE mutation status with limited MMR and/or p53 data—was described in 5 studies [30,34,35,42,47]. POLE-focused analyses without full molecular subclassification were conducted in 3 studies [29,39,44].

#### 3.3.2. POLE Genomic Regions Analyzed

The POLE exonuclease domain, most commonly exons 9–14, was assessed in the majority of studies (14/20). Targeted hotspot sequencing of exons 9, 13, and 14 was performed in 7 studies [30,33,34,37,38,39,44].

#### 3.3.3. Definition of Pathogenic POLE Mutations

All studies restricted analyses to pathogenic or likely pathogenic POLE EDM. Hotspot missense variants—most frequently P286R, S297F, V411L, A456P, and S459F—were consistently included across cohorts. Several studies explicitly excluded variants of uncertain significance, germline POLE mutations, or tumors with concurrent MMRd or p53-abnormal status when assigning the POLE molecular class [30,39].

#### 3.3.4. Sequencing Methodologies

Sanger sequencing was the most frequently used method (11/20 studies), followed by NGS approaches, including targeted panels and comprehensive genomic profiling (7 studies). PCR-based hotspot assays were employed in 3 studies.

#### 3.3.5. Tumor Mutational Burden and Ultramutated Phenotype

Explicit TMB cut-offs were reported in only two studies. One study applied a quantitative mutation index threshold to define ultramutation [38], while another used a TMB ≥10 mutations/Mb as a descriptive threshold [36]. In the remaining studies, the ultramutated phenotype was inferred qualitatively based on predefined pathogenic POLE mutation criteria without numeric TMB thresholds.

### 3.4. Prognostic Impact of POLE Exonuclease-Domain Mutations on Survival Outcomes

Effect size estimates were consistently favorable for POLE-mutant tumors across all comparator definitions; however, the magnitude of the hazard reduction varied by reference group. Comparisons against NSMP-only cohorts yielded more conservative HR estimates, whereas comparisons against pooled non-POLE cohorts, which may include p53-abnormal tumors, tended to show larger effect sizes.

Survival outcomes associated with pathogenic and likely pathogenic POLE EDM were reported across multiple time-to-event endpoints, including OS, DFS, PFS, RFS, and CSS, with effect estimates extracted where available and analyzed according to the predefined analytical plan.

OS outcomes according to POLE EDM status were reported in 8 studies included in the final dataset. Therefore, HR for OS comparing POLE-mutant tumors with the respective comparator groups ranged from 0.12 to 0.91. All reported point estimates favored the POLE-mutant group, although the magnitude of effect and statistical significance varied between studies.

Statistically significant reductions in the risk of death were reported in analyses by Leon-Castillo et al. (2020) and Bosse et al. (2018), with HR of 0.12 (95% CI, 0.02–0.87) and 0.36 (95% CI, 0.18–0.70), respectively [5,46]. Kolehmainen et al. (2020) also reported a significant association, with an OS HR of 0.35 (95% CI, 0.13–0.92) [45]. Non-significant results were reported with wider CIs, including McConechy et al. (2016), Stelloo et al. (2016), Billingsley et al. (2016), Cosgrove et al. (2018), Slomovitz et al. (2025), and Zammarrelli et al. (2022), reflecting limited numbers of death events in POLE-mutant cohorts [29,30,31,32,40,42] (Table 3).

DFS, PFS, RFS, and time to progression (TTP) were reported in 11 studies. Across these analyses, HR comparing POLE-mutant tumors with the respective comparator groups ranged from 0.00 to 0.87, with all point estimates below 1.0.

Statistically significant reductions in the risk of recurrence or progression were reported in five studies: Van Gool et al. (2018) reported an RFS HR of 0.14 (95% CI, 0.001–0.996) [39]; Leon-Castillo et al. (2020) reported 0.08 (95% CI, 0.01–0.58) [5]; Bosse et al. (2018) reported 0.17 (95% CI, 0.05–0.54) [46]; Kolehmainen et al. (2020) reported 0.34 (95% CI, 0.15–0.73) [45]; and Gonzalez-Bosquet et al. (2022) reported 0.33 (95% CI, 0.12–0.91) [33].

The remaining studies reported non-significant associations with wider CIs [29,31,40,42,47]. One study reported no recurrence events in the POLE-mutant group, resulting in a non-estimable HR (Zong et al., 2023) [35] (Table 4).

CSS outcomes according to POLE EDM status were reported in 4 studies. HR for CSS comparing POLE-mutant tumors with the respective comparator groups ranged from 0.00 to 0.32. Statistically significant reductions in cancer-related mortality were observed in analyses by McConechy et al. (2016), reporting an HR of 0.22 (95% CI, 0.02–0.83) [42], and Leon-Castillo et al. (2020), reporting an HR of 0.19 (95% CI, 0.05–0.70) [5]. Zong L (2023) and Billingsley CC (2016) report non-significant associations with wider CIs, including one study with no cancer-specific deaths in the POLE-mutant group [29,35] (Table 5).

### 3.5. Quantitative Synthesis of Overall Survival and Disease Control Outcomes Associated with POLE Exonuclease-Domain Mutations

A random-effects meta-analysis was performed to evaluate the association between POLE EDM and OS. A total of eight studies provided estimable HR and were included in the quantitative synthesis.

**Figure 2:**
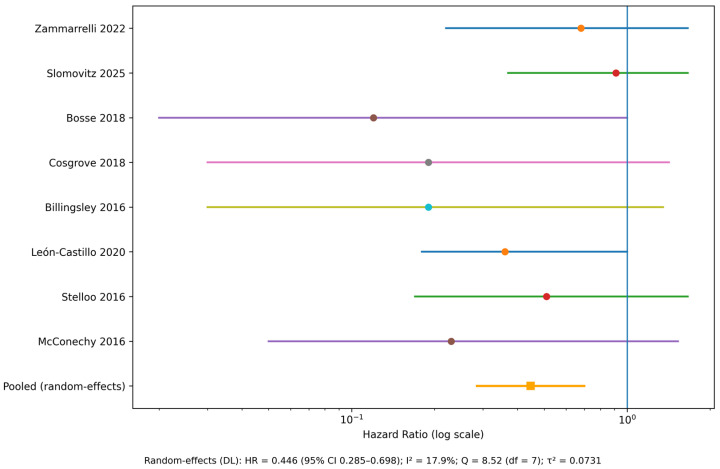
Forest plot (random-effects): OS (POLE-mutant vs. comparator) [5,29,30,31,32,40,42,46]. HR, hazard ratio; CI, confidence interval; DL, DerSimonian–Laird method; OS, overall survival; I^2^, inconsistency index; Q, Cochran’s Q statistic; df, degrees of freedom; τ^2^, between-study variance. Individual study hazard ratios are shown as circles with horizontal lines indicating 95% confidence intervals. The pooled estimate from the random-effects meta-analysis is represented by a square. The vertical line denotes the null effect (HR = 1.0). Hazard ratios are displayed on a logarithmic scale.

The pooled analysis demonstrated a significantly reduced risk of death in POLE-mutant tumors compared with comparator groups, with a pooled HR < 1, indicating a favorable OS associated with POLE mutations (Figure 2). Statistical heterogeneity was low to moderate, as reflected by the I^2^ statistic (17.9%), suggesting acceptable consistency across included studies, despite differences in comparator definitions.

**Figure 3:**
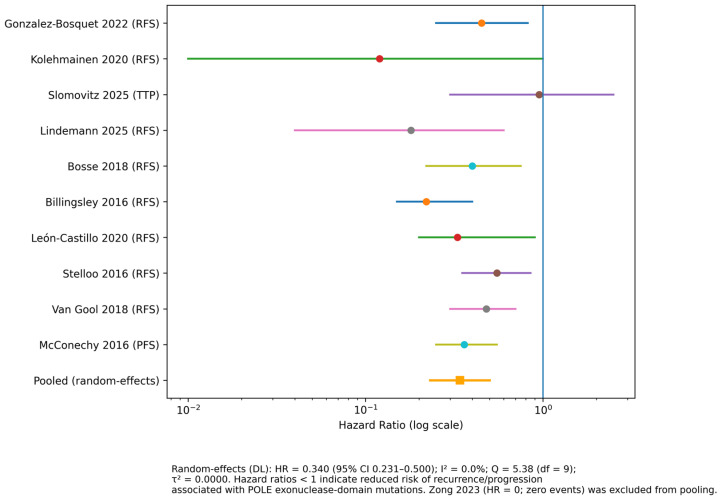
Forest plot (random-effects): DFS/PFS/RFS/TTP (POLE-mutant vs. comparator) [5,29,31,33,39,40,42,45,46,47]. HR, hazard ratio; CI, confidence interval; DL, DerSimonian–Laird method; RFS, recurrence-free survival; PFS, progression-free survival; TTP, time to progression; I^2^, inconsistency index; Q, Cochran’s Q statistic; df, degrees of freedom; τ^2^, between-study variance. Individual study hazard ratios are shown as circles with horizontal lines indicating 95% confidence intervals. The pooled estimate from the random-effects meta-analysis is represented by a square. The vertical line denotes the null effect (HR = 1.0). Hazard ratios are displayed on a logarithmic scale. Studies reporting zero events in the POLE-mutant group were excluded from quantitative pooling.

A separate random-effects meta-analysis was conducted for disease control endpoints (DFS, PFS, RFS, or TTP). Ten studies with estimable HR were included, while one study reporting zero events in the POLE-mutant group was excluded from quantitative pooling and retained for narrative synthesis. The pooled estimate indicated a significantly lower risk of recurrence or progression in POLE-mutant tumors compared with the comparator groups (Figure 3). Between-study heterogeneity was moderate, with Q = 5.8 (df = 9) and I^2^ = 0%, reflecting clinical and methodological differences across studies, including endpoint definitions and comparator selection. One study with zero events in the POLE-mutant group (Zong et al., 2023) was not included in the pooled analysis, per prespecified criteria [35].

### 3.6. Synthesis of Studies with Zero Events or Non-Estimable HR

Several included studies reported zero recurrence or cancer-related death events in the POLE-mutant subgroup, resulting in non-estimable HR and precluding their inclusion in quantitative meta-analyses. In particular, Zong et al. (2023) reported no disease recurrence or cancer-specific deaths among POLE-mutant patients during follow-up, yielding an HR of 0.00 with undefined precision [35]. Similar patterns of absent events were observed in POLE-mutant cohorts in smaller institutional series and in subgroup analyses embedded within larger molecular classification studies.

These zero-event cohorts were predominantly composed of patients with early-stage disease, often enriched for high-grade histology, and frequently treated with standard surgical management, with or without adjuvant therapy, according to institutional or trial-specific protocols.

Although such studies could not be included in pooled HR meta-analyses due to statistical non-assessability, they provided clinically relevant prognostic information by consistently demonstrating exceptionally favorable outcomes in POLE-mutant EC. Their findings complement the quantitative results and reinforce the prognostic profile observed across studies with estimable effect measures.

### 3.7. Risk of Bias

The risk of bias across the included studies was low to moderate (as detailed in Table S5 in the Supplementary Materials). The lowest risk was observed in the classification of exposure (POLE mutation status) and in the measurement of outcomes, reflecting the use of validated molecular techniques and objective survival endpoints. Moderate risk of bias due to confounding and participant selection was common, primarily related to retrospective study designs and incomplete adjustment for prognostic variables. No study was judged to be at critical risk of bias in any ROBINS-I domain.

## 4. Discussion

This systematic review and meta-analysis demonstrates that pathogenic and likely pathogenic POLE EDMs are consistently associated with a favorable prognosis in EC. Across individual studies and pooled analyses, POLE-mutant tumors showed reduced hazards for OS, DFS/PFS/RFS, and CSS, with HR uniformly below 1.0. Random-effects meta-analyses confirmed this association for both OS and disease control endpoints, with low-to-moderate between-study heterogeneity, indicating acceptable consistency despite differences in study design and comparator definitions.

In addition, several studies reported zero recurrences or cancer-related deaths among POLE-mutant cohorts during follow-up, precluding quantitative pooling but providing compelling qualitative evidence of an exceptionally favorable clinical course. Taken together, these findings establish POLE EDM as a robust prognostic marker across diverse clinical settings, encompassing early-stage, high-risk, and selected advanced or recurrent EC populations.

The findings of the present study align closely with high-quality evidence establishing the prognostic superiority of molecular classification over histology alone in EC. The PORTEC-3 trial, a prospective clinical study with molecular annotation, demonstrated that molecular subgroups identified by integrated genomic profiling provide markedly different survival outcomes within high-risk EC. Specifically, the 5-year RFS rates were 98% for POLE-mutant tumors, 72% for mismatch repair-deficient tumors, 74% for NSMP tumors, and 48% for p53-abnormal tumors (*p* < 0.001), underscoring the exceptionally favorable course of POLE-mutant disease relative to other subtypes [5,11].

Although “multiple-classifier” endometrial carcinomas, such as tumors harboring concurrent POLE mutations with MMR deficiency or p53 abnormalities, are increasingly recognized in the molecular classification era, they appear to be rare events and were not reported in the studies included in this analysis; their limited representation and inconsistent handling in the literature underscore an important methodological challenge, as the prognostic dominance of POLE alterations in such contexts remains incompletely defined and warrants standardized reporting in future molecularly stratified investigations [48,49]. On the other hand, a recent study reports no recurrences in POLEmut-p53abn and POLE mut, suggesting that POLE can dominate the phenotype [48].

These results were supported by broader molecular epidemiology, including TCGA classification, which consistently identified the POLE-ultramutated subgroup as an outlier with very low risk of progression and death, despite frequent high-grade histology. Systematic reviews and meta-analyses have similarly reported that POLE-mutated tumors exhibit significantly better progression-free and OS than other molecular types [50,51,52]. In addition, other observational and cohort studies have demonstrated the robustness of the molecular classification in refining prognosis beyond traditional clinicopathologic parameters. Molecular stratification has been shown to more accurately predict recurrence and survival patterns after relapse than histologic grade or stage alone, reinforcing the concept that POLE-mutant tumors represent a distinct biologic entity with an intrinsically favorable outcome [53].

Studies reporting zero recurrences or cancer-related deaths in POLE-mutant EC cohorts reflected the intrinsic biology of the POLE molecular subtype, rather than a statistical artifact. From a methodological standpoint, zero-event data yield non-estimable HRs. They cannot be robustly included in quantitative meta-analyses without compromising statistical validity, justifying their exclusion from pooled estimates. Nevertheless, their inclusion in the narrative was clinically informative, as they substantiate the exceptionally favorable prognosis of POLE-mutant EC and provide important context for the quantitative survival analyses.

The favorable prognosis associated with POLE EDM in EC is biologically plausible and well supported by mechanistic evidence. Pathogenic mutations affecting the proofreading domain of DNA polymerase epsilon led to defective replication fidelity, resulting in an exceptionally high burden of somatic single-nucleotide variants and an ultramutated genomic phenotype [54,55]. POLE-mutant ECs consistently exhibited extremely high tumor mutational burden (TMB), often exceeding conventional thresholds for hypermutated tumors, thereby generating an extensive repertoire of neoantigens that can elicit a robust antitumor immune response [5,39,40,56].

Multiple translational and clinicopathologic studies have demonstrated that POLE-mutant tumors are characterized by dense tumor-infiltrating lymphocytes, particularly CD8+ cytotoxic T cells, along with increased expression of immune-related genes and interferon-γ-associated signatures [39,44,46,57,58]. TILs should be interpreted in two distinct contexts in POLE-mutant endometrial cancer. First, increased TIL density represents a downstream biological consequence of the ultramutated genotype, mediating enhanced antitumor immune surveillance and contributing mechanistically to the favorable prognosis observed in POLE-mutant tumors. Second, beyond this mechanistic role, TILs have also been proposed as a potential histopathological surrogate marker for POLE mutation status, particularly in settings where molecular testing is unavailable. Previous systematic and cohort-based analyses have shown that a TIL-high phenotype demonstrates moderate discriminatory accuracy for identifying POLE-mutant tumors, particularly when mismatch repair-deficient cases are excluded, suggesting potential utility as a histopathological surrogate within TCGA- or ProMisE-aligned risk assessment frameworks when molecular testing is not readily available [41,59,60]. This immunogenomic context provides a compelling explanation for the effective immune-mediated tumor control, translating clinically into very low recurrence rates and excellent survival, even in tumors with adverse histologic features, such as high-grade tumors. The survival patterns observed in the present study, consistently reduced HRs for OS, DFS/PFS/RFS, and CSS, as well as the presence of zero-recurrence cohorts, are therefore concordant with this established biological framework and reinforce the concept that POLE-mutant EC represents a distinct, immunologically advantaged molecular subtype with intrinsically favorable outcomes [5,38].

Pooled survival estimates in this analysis provided quantitative support for the current shift toward molecularly guided treatment individualization in EC, where POLE EDM identified a subgroup with sufficiently favorable outcomes to justify treatment de-escalation in appropriately selected early-stage patients. Pooling across heterogeneous comparator groups may introduce bias in the magnitude of the estimated effect. Specifically, comparisons with pooled non-POLE cohorts that include p53-abnormal tumors are expected to overestimate the relative prognostic advantage of POLE-mutant tumors, whereas comparisons restricted to NSMP or MMRd cohorts are likely to yield more conservative estimates. Importantly, despite these differences in magnitude, the direction of effect was uniformly favorable across all comparator definitions, supporting the robustness of POLE mutation as a prognostic marker. From a clinical perspective, comparator heterogeneity reflects the historical evolution of molecular classification in endometrial cancer. Current ESGO/ESTRO/ESP guidelines emphasize molecular subgroup-specific risk stratification; therefore, effect estimates derived from pooled non-POLE comparators should be interpreted cautiously when applied to contemporary, molecularly stratified clinical decision-making [61,62]. The integration of molecular subtype into staging and prognostic stratification was further reinforced by the FIGO 2023 staging system, which recognized that POLEmut and p53-abnormal status can modify prognostic grouping in early-stage disease [13]. In parallel, de-escalation is being prospectively tested in molecular profile-based adjuvant strategies (PORTEC-4a), reflecting the field’s move from histology-driven intensification toward risk-adapted omission of adjuvant treatment in favorable molecular profiles [51,63]. Therefore, while the favorable survival outcomes observed in POLE-mutant endometrial cancer are robust, they should be interpreted as reflecting an integrated molecular–immune phenotype rather than the isolated effect of POLE mutation status alone.

Within this evolving framework, the present meta-analysis strengthens the evidentiary basis for these recommendations by confirming, across independent cohorts and endpoints, consistently reduced hazards for OS and recurrence-related outcomes in POLEmut EC, supporting efforts to avoid overtreatment when molecular classification is available.

Although statistical heterogeneity was low to moderate, this should not be interpreted as conceptual homogeneity, as comparator definitions varied substantially across studies. The consistent direction of effect across heterogeneous comparators likely reflects the strong biological signal associated with POLE mutations, whereas the magnitude of the pooled estimates may be influenced by the prognostic profile of the reference groups.

Current ESGO/ESTRO/ESP guidelines emphasize treatment individualization based on molecular classification, yet the inconsistent reporting of therapy by POLE status in the available literature constrains clinically actionable interpretation; notably, molecular analyses from the PORTEC-3 trial demonstrated excellent outcomes for POLE-mutant tumors across treatment arms, supporting the ongoing shift toward risk-adapted management and treatment de-escalation in appropriately selected patients [5,61].

Key strengths of this study include the a priori PROSPERO registration, adherence to PRISMA 2020, use of ROBINS-I for structured risk-of-bias assessment, and the performance of separate random-effects meta-analyses by endpoint, with explicit handling of zero-event studies. Between-study heterogeneity was expected and primarily reflects differences in clinical setting, comparator definitions, molecular testing strategies, and endpoint definitions across cohorts. Importantly, heterogeneity was low to moderate in pooled analyses and did not materially affect the direction or robustness of the observed associations, supporting the validity of the conclusions.

This study had several limitations that should be acknowledged. Most included studies were retrospective, introducing potential selection bias and residual confounding despite multivariable adjustment. An important limitation is that most included studies did not systematically assess or adjust for TILS density in survival analyses. The absolute number of POLE-mutant cases was small, reflecting the low prevalence of this molecular subtype and limiting statistical power in some analyses. In addition, TMB was not uniformly assessed or standardized, precluding quantitative evaluation of TMB thresholds across studies.

Future research should focus on prospective, molecularly stratified randomized trials, the harmonization and validation of clinically relevant TMB cut-offs, and dedicated prospective studies of treatment de-escalation in POLE-mutant EC to refine risk-adapted therapeutic strategies. Also, these findings suggest that POLE EDMs define a subgroup with an exceptionally favorable prognosis, supporting molecular risk stratification and the avoidance of overtreatment in guideline-concordant settings. In advanced-stage disease, current evidence remains insufficient, necessitating individualized, multidisciplinary decision-making. The hypermutated and immunogenic profile of POLE-mutant tumors highlights their biological relevance for immunotherapeutic strategies, despite comparatively limited clinical evidence relative to dMMR/MSI-H cancers.

## 5. Conclusions

This research demonstrates that pathogenic and likely pathogenic POLE EDMs are associated with a highly favorable prognosis in EC. Across individual studies and pooled analyses, POLE-mutant tumors showed reduced HR for OS, DFS/PFS/RFS, and CSS, with all reported HRs favoring the POLE-mutant subgroup. Random-effects meta-analyses confirmed a significant survival benefit for both OS and disease control outcomes, with low-to-moderate heterogeneity, supporting the robustness of these findings across heterogeneous clinical settings. Notably, several studies reported no recurrences or cancer-related deaths among patients with POLE-mutant EC. These observations were consistent across cohorts that included early-stage, high-grade, and selected high-risk or advanced cases. The favorable outcomes observed in this analysis are concordant with established molecular classification frameworks and reflect the distinct biological and immunogenic characteristics of POLE-mutant tumors. These results establish POLE-EDM status as a strong, reproducible prognostic marker in EC, reinforcing its central role in molecular risk stratification and contemporary clinical management.

## Figures and Tables

**Table 1:** Main characteristics of the included studies.

First Author, Year	Country	Study Design	Study Period	Total Patients (*n*)	POLE Mutant (*n*, %)	Disease Stage	Histology	Molecular Classification	Comparator Used	Primary Survival Outcome
Aksahin E, 2025 [41]	Turkey	Retrospective cohort study	2000–2022	114	8 (7.0%)	Predominantly early-stage (FIGO I–II)	Endometrioid	TCGA-aligned (POLE, MMRd, p53abn, NSMP)	DFS, OS	Non-POLE (pooled)
McConechy M, 2016 [42]	Canada	Retrospective cohort study	1983–2013	406	39 (9.6%)	Predominantly early-stage	Mixed histology (endometrioid predominant)	Partial (POLE-focused molecular analysis)	PFS, DFS, OS, CSS	POLE wild-type (wt)
Van Gool I, 2018 [39]	Netherlands	Retrospective analysis of RCT cohort	1980–1990	245	16 (6.5%)	Stage I only	Endometrioid	POLE-mutant vs. POLE wt (restricted cohort)	Recurrence-free survival	POLE wt
Stelloo E, 2016 [40]	Netherlands	Retrospective analysis of RCT cohorts (PORTEC-1/2)	1990–2006	834	49 (5.9%)	Early-stage (FIGO I–II)	Endometrioid	TCGA surrogate (POLE, MSI, p53abn, NSMP)	RFS, DSS, OS	Molecular subgroups
Joe S, 2023 [37]	Korea	Retrospective cohort study	2013–2018	183	29 (15.9%)	Early-stage (FIGO I–II)	Predominantly endometrioid	TCGA surrogate (POLE, MMRd, p53abn, NSMP)	RFS, OS	Molecular sub-groups
Andrade DAP, 2024 [43]	Brazil	Prospective cohort study	2020–2022	114	18 (15.8%)	All stages	Mixed histology	ProMisE	OS, PFS	Molecular sub-groups
He D, 2020 [34]	China	Retrospective cohort study	2011–2016	426	38 (8.9%)	Predominantly early-stage	Mixed histology	Partial (POLE + MMR/p53 IHC)	OS, PFS	POLE wt
Zong L, 2023 [35]	China	Retrospective cohort study	2010–2018	335	42 (11.8%)	High-grade (all stages)	High-grade ECs	TCGA surrogate	RFS, DSS	Molecular sub-groups
Leon-Castillo A, 2020 [5]	Multi-national	Translational analysis of randomized phase III trial	NR	410	51 (12.4%)	High-risk	Mixed histology	TCGA surrogate	RFS, OS	Molecular sub-groups
Han KH, 2024 [38]	Korea	Retrospective cohort study	2014–2018	161	19 (11.8%)	All stages (FIGO I–IV; FIGO 2023 applied retrospectively)	Mixed (endometrioid predominant)	TCGA surrogate (POLE, MMRd, p53abn, NSMP)	PFS, OS, DSS	Molecular sub-groups
Leon-Castillo A, 2022 [44]	Denmark	Retrospective population-based cohort study	2005–2012	367	38 (10.4%)	Stage I–III only (high-grade)	High-grade EC only	TCGA surrogate	Recurrence, OS, DSS	Molecular sub-groups
Billingsley CC, 2016 [29]	United States	Retrospective cohort study	NR	72	7 (9.7%)	Stage I–IV	Endometrioid only (grade 3)	Partial (POLE-focused)	RFS, OS	POLE wt
Cosgrove CM, 2018 [30]	United States	Retrospective cohort study (GOG-210)	2003–2007	982	39 (4.0%)	All stages	Endometrioid	TCGA (CNS, MMRd, CNA, POLE)	PFS, ECS, OS	Non-POLE (pooled)
Bosse T, 2018 [46]	Multi-center	Retrospective cohort study	NR	381	49 (12.9%)	All stages	Endometrioid (grade 3)	TCGA surrogate	OS, RFS	NSMP
Lindemann K, 2025 [47]	Norway	Retrospective cohort study	2006–2017	360 (advanced/ recurrent)	11 (~3.1%)	Advanced (III–IV) and recurrent	Mixed histology	ProMisE	Time to recurrence (TTR), CSS	Molecular sub-groups
Slomovitz BM, 2025 [31]	United States	Retrospective cohort study	2011–2023	1139	12 (1.0%)	All stages (advanced/recurrent enriched)	Mixed histology	TCGA/ProMisE	Time to next treatment (TTNT), OS	NSMP
Guo Q, 2024 [36]	China	Retrospective cohort study	2020–2022	331	47 (14.2%)	All stages	Predominantly endometrioid	TCGA	DFS	Molecular sub-groups
Zammarrelli WA, 2022 [32]	United States	Retrospective cohort study	2014–2020	75	24 (32.0%)	Stage I only (grade 3)	Endometrioid	TCGA	PFS, OS	CN-H
Kolehmainen A, 2020 [45]	Finland	Retrospective cohort study	2007–2012	515	37 (7.2%)	All stages (early-stage predominant)	Predominantly endometrioid	Trans-PORTEC	Cancer- related mortality	Molecular sub-groups
Gonzalez-Bosquet J, 2022 [33]	United States (TCGA)	Retrospective molecular cohort	NR	192	28 (14.6%)	All stages	Endometrioid	TCGA	PFS	Reference a favorable group

EC—endometrial cancer; FIGO—International Federation of Gynecology and Obstetrics; RCT—randomized controlled trial; TCGA—The Cancer Genome Atlas; ProMisE—Proactive Molecular Risk Classifier for Endometrial Cancer; PORTEC—Post Operative Radiation Therapy in Endometrial Carcinoma; GOG—Gynecologic Oncology Group; POLE—DNA polymerase epsilon exonuclease domain; wt—wild-type; MMRd—mismatch repair–deficient; MSI—microsatellite instability; p53abn—abnormal p53; NSMP—no specific molecular profile; CNA—copy number altered; CNS—copy number stable; CN-H—copy number–high; DFS—disease-free survival; PFS—progression-free survival; OS—overall survival; CSS—cancer-specific survival; DSS—disease-specific survival; RFS—recurrence-free survival; ECS—endometrial cancer–specific survival; TTR—time to recurrence; TTNT—time to next treatment; NR—not reported; IHC—immunohistochemistry.

**Table 2:** Molecular testing and molecular classification characteristics of the included studies.

First Author, Year	POLE Mutant Cases (*n*)	Molecular Subgroup Reported	POLE Region Tested	POLE Pathogenicity Definition	Sequencing Method	Ultramutated/ TMB Cutoff
Aksahin E, 2025 [41]	8	POLE-mut 5–7%; MMR-d 43–47%; p53-mut ~5–10%	Exons 9–14	Pathogenic somatic mutations in POLE exonuclease domain (including duplication c.1368_1370dup, p.T457dup) have been reported.	Sanger	NR
McConechy M, 2016 [42]	39	Partial molecular classification reported	Exons 9–14	Hotspot and non-hotspot variants; germline POLE excluded	Sanger	NR
Van Gool I, 2018 [39]	16	Only POLE-mutant vs. POLE wild-type analyzed	Exons 9, 13, and 14	Pathogenic POLE EDM mutations; concurrent p53 mutation or MMR deficiency excluded	Sanger	NR
Stelloo E, 2016 [40]	49	p53-mutant: 9%; MSI: 26%; POLE-mutant: 6%; NSMP: remainder	Exons 9 and 13	Hotspot pathogenic somatic POLE EDM.	Sanger	NR
Joe S, 2023 [37]	29	POLEmut 15.9%; MMR-D 29.0%; p53abn 8.7%; NSMP 46.4%	Exons 9, 13, and 14	Pathogenic POLE EDM hotspot mutations (P286R, S297F, V411L, A456P, S459F)	PCR	NR
Andrade DAP, 2024 [43]	18	ProMisE: POLEmut 15.8%, MMRd 28.1%, p53abn 10.5%, NSMP 45.6%	Exons 9–14	POLE EDM pathogenic variants (as per ProMisE methodology; hotspot sequencing)	Sanger	NR
He D, 2020 [34]	38	Partial (POLE mutation + MMR and p53 IHC status reported)	Exons 9, 13, and 14	POLE EDM missense variants (incl. P286R, V411L, Q453R; novel variants F274L, G420D, V460A)	Sanger	NR
Zong L, 2023 [35]	42	TCGA surrogate: POLEmut 11.8%, MMRd 29.9%, p53abn 36.1%, NSMP 22.2%	Exons 9–14	Validated pathogenic POLE EDM mutations (P286R, V411L, S297F, A456P, S459F)	Sanger	NR
Leon-Castillo A, 2020 [5]	51	p53abn 22.7%; POLE-mut 12.4%; MMRd 33.4%; NSMP 31.5%	Exons 9–14 (EDM)	Pathogenic POLE EDM mutations are considered causative of the ultramutated phenotype (predefined criteria)	NGS	NR
Han KH, 2024 [38]	19	TCGA surrogate: POLEmut 11.8%; MMRd 28.6%; p53abn 23.3%; NSMP 46.0%	Exons 9, 13, and 14 (hotspots)	Pathogenic hotspot POLE EDM mutations (P286R, S297F, V411L, A456P, S459F)	PCR	≥6 copies/20 μL or ≥0.3% mutation index
Leon-Castillo A, 2022 [44]	38	Yes	Exons 9, 13, and 14	Pathogenic POLE EDM mutations per Leon-Castillo et al. criteria (ultramutated phenotype)	NGS	NR
Billingsley CC, 2016 [29]	7	POLE only	Exonuclease domain residues 268–471	Somatic missense mutations in the POLE exonuclease (proofreading) domain	Sanger	NR
Cosgrove CM, 2018 [30]	39	CNS, MMR-deficient, CNA, POLE-mutant	Exons 9, 13, and 14	Pathogenic POLE EDM mutations; POLE class assigned only if MMR-proficient and copy-number stable	Sanger	NR
Bosse T, 2018 [46]	49	POLE 12.9%; MMRd 36.2%; p53abn 20.7%; NSMP 30.2%	Exons 9–14	Pathogenic POLE EDM hotspot mutations; TCGA-ultramutated definition	Sanger	NR
Lindemann K, 2025 [47]	11	POLE + p53 and MMR protein expression assessed	NR	Known pathogenic POLE EDM mutations (e.g., P286R, V411L, Q453R)	Sanger	NR
Slomovitz BM, 2025 [31]	12	Advanced: POLE 4%, MMRd 25%, p53abn 44%, NSMP 26%; Recurrent: POLE 1%, MMRd 21%, p53abn 48%, NSMP 28%	NR	Pathogenic POLE mutations according to ESMO/ProMisE criteria	Sanger	NR
Guo Q, 2024 [36]	47	POLEmut 1%; MSI-H 22%; TP53mut 47%; NSMP 31%	NR	POLE EDM pathogenic/likely pathogenic (TCGA/ProMisE-aligned)	NGS	High TMB ≥10 mutations/Mb (descriptive)
Zammarrelli WA, 2022 [32]	24	POLEmut 14.2%; dMMR 23.9%; p53abn 17.2%; NSMP 44.7%	NR	Pathogenic/likely pathogenic POLE EDM (WHO/TCGA molecular classification)	NGS	NR (explicit numeric cutoff not applied)
Kolehmainen A, 2020 [45]	37	POLE 32%; MSI/MMRd 35%; CN-H/p53-abn 20%; CN-L/NSMP 13%	NR	POLE EDM hotspot mutation by MSK-IMPACT	NGS	NR
Gonzalez-Bosquet J, 2022 [33]	28	NSMP 218; POLE 37; MMR-D 191; p53abn 69	Exons 9, 13, and 14	Hotspot POLE EDM mutations (P286R, S297F, V411L, A456P)	Sanger	NR

POLE—DNA polymerase epsilon; EDM—exonuclease (proof-reading) domain; MMRd—mismatch repair–deficient; dMMR—deficient mismatch repair; MSI—microsatellite instability; MSI-H—microsatellite instability–high; p53abn—abnormal p53 expression; TP53mut—TP53 gene mutation; NSMP—no specific molecular profile; CNS—copy number stable; CNA—copy number altered; CN-H—copy number–high; CN-L—copy number–low; ProMisE—Proactive Molecular Risk Classifier for Endometrial Cancer; TCGA—The Cancer Genome Atlas; ESMO—European Society for Medical Oncology; WHO—World Health Organization; MSK-IMPACT—Memorial Sloan Kettering–Integrated Mutation Profiling of Actionable Cancer Targets; IHC—immunohistochemistry; PCR—polymerase chain reaction; NGS—next-generation sequencing; TMB—tumor mutational burden; Mb—megabase; NR—not reported.

**Table 3:** OS: HR for POLE-mutant versus comparator tumors.

First Author, Year	Comparator	HR	95% CI
McConechy M, 2016 [42]	POLE wt	0.69	0.22–1.67
Stelloo E, 2016 [40]	NSMP	0.91	0.37–1.67
Leon-Castillo A, 2020 [5]	Reference	0.12	0.02–0.87
Billingsley CC, 2016 [29]	POLE wt	0.19	0.03–1.42
Cosgrove CM, 2018 [25]	POLE wt	0.19	0.03–1.35
Bosse T, 2018 [46]	NSMP	0.36	0.18–0.7
Slomovitz BM, 2025 [31]	NSMP	0.52	0.17–1.66
Zammarrelli WA, 2022 [32]	Reference	0.23	0.05–1.53

HR < 1 indicates reduced risk of events in POLE-mutant tumors relative to the comparator group.

**Table 4:** Disease-Free/Progression-Free/Recurrence-Free Survival.

First Author, Year	Outcome Reported	Comparator	HR	95% CI
McConechy M, 2016 [42]	PFS	POLE wt	0.48	0.10–1.48
Van Gool I, 2018 [39]	RFS	POLE wt	0.14	0.001–0.996
Stelloo E, 2016 [40]	RFS	NSMP	0.87	0.12–6.53
Zong L, 2023 [35]	DFS	NSMP	0.00	0.00–1.7
Leon-Castillo A, 2020 [5]	RFS	Reference	0.08	0.01–0.58
Billingsley CC, 2016 [29]	RFS	POLE wt	0.37	0.09–1.55
Bosse T, 2018 [46]	RFS	NSMP	0.17	0.05–0.54
Lindemann K, 2025 [47]	RFS	Reference	0.32	0.08–1.32
Slomovitz BM, 2025 [31]	TTP	NSMP	0.50	0.21–1.21
Kolehmainen A, 2020 [45]	RFS	Reference	0.34	0.15–0.73
Gonzalez-Bosquet J, 2022 [33]	RFS	POLE wt	0.33	0.12–0.91

**Table 5:** Cancer-specific survival (CSS).

First Author, Year	Comparator	HR	95% CI
McConechy M, 2016 [42]	POLE wt	0.22	0.02–0.83
Zong L, 2023 [35]	NSMP	0.00	0.00–1.9
Leon-Castillo A, 2020 [5]	Reference	0.19	0.05–0.7
Billingsley CC, 2016 [29]	POLE wt	0.32	0.08–1.32

## Data Availability

The data supporting this study are available from the corresponding author upon request.

## Supplementary Materials

The following supporting information can be downloaded at https://www.mdpi.com/article/10.3390/cancers18040597/s1, Table S1: Excluded studies; Table S2: Full search strategies for study search; Table S3: ROBINS-I for Risk of Bias Assessment; Table S4: Extended study characteristics; Table S5: Risk of Bias Ratings.

## Abbreviations

The following abbreviations are used in this manuscript:

CI	Confidence interval
CSS	Cancer-specific survival
DFS	Disease-free survival
EC	Endometrial cancer
EDM	Exonuclease-domain mutation
ESGO	European Society of Gynaecological Oncology
ESP	European Society for Medical Oncology
ESTRO	European Society for Medical Oncology
FIGO	International Federation of Gynecology and Obstetrics
HR	Hazard ratio
MMR	Mismatch repair
MSI-H	Microsatellite instability—high
NGS	Next-generation sequencing
NSMP	No specific molecular profile
OS	Overall survival
PCR	Polymerase chain reaction
PFS	Progression-free survival
POLE	DNA polymerase epsilon
PORTEC	Post-Operative Radiation Therapy in Endometrial Cancer trial
PRISMA	Preferred Reporting Items for Systematic Reviews and Meta-Analyses
ProMisE	Proactive Molecular Risk Classifier for Endometrial Cancer
PROSPERO	International Prospective Register of Systematic Reviews
RCT	Randomized clinical trial
RFS	Recurrence-free survival
ROBINS-I	Risk Of Bias in Non-randomized Studies of Interventions
TCGA	The Cancer Genome Atlas
TILs	Tumor-infiltrating lymphocytes
TTNT	Time to next treatment
TTP	Time to progression
TTR	Time to recurrence
TMB	Tumor mutational burden
WES	Whole-exome sequencing
