# Supplementary material for: Prognostic Impact of POLE Exonuclease-Domain Mutations in Endometrial Cancer: A Systematic Review and Meta-Analysis
Source: Cancers (Basel). 2026 Feb 11;18(4):597. doi: 10.3390/cancers18040597 (PMC12939538; doi:10.3390/cancers18040597)
Supplement: Supplementary file 1 [file cancers-18-00597-s001.zip › cancers-4101420-supplementary.pdf]

cancers

MDPI

Supplementary Materials: Prognostic Impact of POLE Exonuclease-Domain Mutations in Endometrial Cancer: A Systematic Review and Meta-Analysis

Ioana Hurmuz, Robert Barna, Aura Jurescu, Bianca Natarâș, Dorela-Codruța Lăzureanu, Iuliana-Anamaria Trăilă, Alexandru-Marius Furău, Sorina Tăban and Alis Dema

Table S1. Excluded studies.

Study	Title	Reason for Exclusion
**Léon-Castillo A., 2019**	Prognostic relevance of the molecular classification in high-risk endometrial cancer: analysis of the PORTEC-3 trial	Conference abstract only; no extractable HRs or 95% confidence intervals (CIs) for POLE mutations
**Zhang Q., 2021**	Clinicopathologic characteristics of distinct molecular subtypes of endometrial carcinoma in Chinese population	No HR or CIs reported for POLE mutations; survival reported only as proportions.
**Rios-Doria E., 2022**	Molecular classification of endometrial carcinomas: a single-institution review	No HR or CIs were explicitly reported for POLE mutations; HRs were reported only for the CN-H subgroup.
**Quispe EM, 2023**	Prognostic classification of endometrial cancer according to transcriptomic-based immunophenotype	Survival outcomes were reported descriptively only; no hazard ratios or 95% confidence intervals were provided for POLE mutations, and no explicit comparator group was suitable for quantitative synthesis.
**Moreira I, 2023**	Molecular classification of endometrial carcinoma: Analysis of a multicenter Portuguese cohort	Conference abstract only (no full-text available); hazard ratios with 95% confidence intervals for POLEmut vs comparator not reported, and insufficient data to derive them
**Xhindoli L, 2023**	The impact of molecular classes on oncologic outcome of endometrial cancer: a prospective analysis from a tertiary referral center	Conference abstract only (no full-text available); HR with 95% CIs not reported and insufficient data to derive them; internal inconsistency in subgroup counts (POLEmut n=26 vs recurrence data 0/28)
**Siegenthaler F, 2025**	Added prognostic value of sentinel lymph node mapping in endometrial cancer to molecular subgroups	Abstract-only/insufficient reporting for effect extraction: HRs/95% CIs not reported (despite Cox regression being mentioned), no extractable POLEmut-versus-comparator survival effect estimate for pooling.
**Cohen A, 2022**	Overall survival with or without adjuvant radiotherapy among different molecular subtypes of endometrial cancer	Abstract-only/insufficient reporting for effect extraction: HRs/95% CIs not reported.
**Dow J et al., 2024**	Prognostic value of PORTEC-3 molecular markers by disease risk in a real-world early endometrial cancer cohort	No extractable effect estimates: the study reports recurrence-free survival trends by molecular subgroup but does not provide HR, CIs, or sufficient Kaplan–Meier data to derive time-to-event effect measures for POLE-mutated versus comparator .
**Gonzalez-Bosquet J et al., 2025**	ECPPF stratification identifies occult high-risk subgroups in stage I, grade 1 or 2, ≤50% invasive endometrial cancer	Ineligible prognostic framework: Although POLE mutations are reported, the study evaluates a composite molecular classifier (ECPPF) rather than the independent prognostic effect of POLE exonuclease-domain mutations, and does not report hazard ratios or confidence intervals suitable for meta-analysis.

Table S2. Full search strategies for study search.

**PubMed (MEDLINE)**	**("Endometrial Neoplasms"[Mesh] OR endometrial cancer[tiab] OR endometrial carcinoma[tiab] OR uterine cancer[tiab])<br>AND<br>(POLE[tiab] OR "DNA Polymerase II"[Mesh] OR polymerase epsilon[tiab] OR "DNA polymerase epsilon"[tiab])<br>AND<br>(exonuclease[tiab] OR proofreading[tiab] OR "exonuclease domain"[tiab] OR ultramutated[tiab])<br>AND<br>(prognosis[Mesh] OR prognosis[tiab] OR survival[tiab] OR "overall survival"[tiab] OR "disease-free survival"[tiab] OR "progression-free survival"[tiab])<br>NOT<br>(review[pt] OR case reports[pt])**
**Embase**	('endometrial cancer'/exp OR 'endometrial carcinoma':ab,ti OR 'uterine cancer':ab,ti)<br>AND<br>('dna polymerase epsilon'/exp OR pole:ab,ti OR 'polymerase epsilon':ab,ti)<br>AND<br>(exonuclease:ab,ti OR 'exonuclease domain':ab,ti OR ultramutated:ab,ti)<br>AND<br>(prognosis/exp OR survival/exp OR survival:ab,ti OR 'overall survival':ab,ti OR 'disease free survival':ab,ti OR 'progression free survival':ab,ti)<br>NOT<br>(review:it OR 'case report':it)
**Web of Science**	TS=("endometrial cancer" OR "endometrial carcinoma" OR "uterine cancer")<br>AND TS=(POLE OR "polymerase epsilon" OR "DNA polymerase epsilon")<br>AND TS=(exonuclease OR "exonuclease domain" OR ultramutated)<br>AND TS=(prognosis OR survival OR "overall survival" OR "disease-free survival" OR "progression-free survival")<br>NOT DT=(Review)

Table S3. ROBINS-I for Risk of Bias Assessment.

Domain	Criteria	Rating of Bias Risk
**1. Bias due to confounding**	This domain evaluated whether studies adequately addressed potential confounding factors that could influence the association between POLE exonuclease-domain mutation status and survival outcomes. Key confounders considered included age, FIGO stage, histological subtype, tumor grade, lymphovascular space invasion (LVSI), adjuvant treatment, and molecular subgroup (MMRd, p53-abnormal, NSMP). Studies were rated at low risk if multivariable models adjusted for major prognostic factors or if the study design inherently minimized confounding.	a. Low risk: All major confounders appropriately accounted for.<br>b. Moderate risk: Partial adjustment or unclear handling of confounders.<br>c. High risk: No adjustment or major confounders unaddressed.
**2. Bias in the selection of participants**	This domain assessed whether the study population was clearly defined and representative of the target population of patients with endometrial cancer. We evaluated inclusion and exclusion criteria, selection based on molecular testing availability, and whether excluding specific subgroups (e.g., advanced or recurrent disease) could introduce selection bias.	a. Low risk: Clearly defined and representative population.<br>b. Moderate risk: Some selection criteria are unclear or restrictive.<br>c. High risk: Highly selected or non-representative population.

**3. Bias in classification of exposure (POLE status)**	This domain examined the accuracy and consistency of POLE exonuclease-domain mutation assessment, including the genomic regions tested, the sequencing methodologies (Sanger, NGS, WES), and the variant classification (pathogenic/likely pathogenic). Studies using validated methods and clearly defined pathogenic criteria were considered at low risk.	a. Low risk: Validated molecular methods and clear mutation definitions.<br>b. Moderate risk: Limited reporting of testing methods or variant classification.<br>c. High risk: Unclear or potentially misclassified POLE status.
**4. Bias due to deviations from intended exposure**	This domain evaluated whether deviations from the intended exposure occurred after classification, such as differential treatment allocation or post-hoc reclassification of molecular subgroups that could influence outcomes. Given the prognostic (non-interventional) nature of the exposure, this domain was generally considered low risk unless there were apparent post-classification deviations.	a. Low risk: No evidence of deviations affecting outcomes.<br>b. Moderate risk: Minor or unclear deviations.<br>c. High risk: Substantial deviations likely influencing outcomes.
**5. Bias due to missing data**	This domain assessed the extent and handling of missing data for key variables, including survival outcomes, molecular classification, and covariates. Studies were rated as higher risk if missing data were substantial, unreported, or inadequately handled.	a. Low risk: Minimal missing data or appropriate handling.<br>b. Moderate risk: Some missing data with unclear handling.<br>c. High risk: Extensive or inadequately addressed missing data.
**6. Bias in measurement of outcomes**	This domain evaluated how survival outcomes (OS, PFS, DFS, CSS) were measured, including the use of standardized definitions, adequate follow-up duration, and objective ascertainment of outcomes. As survival outcomes are generally objective, this domain was typically low risk unless outcome definitions or follow-up were unclear.	a. Low risk: Objective and consistently defined outcomes.<br>b. Moderate risk: Some ambiguity in outcome definitions or follow-up.<br>c. High risk: Poorly defined or inconsistently measured outcomes.
**7. Bias in the selection of the reported result**	This domain assessed whether selective reporting of outcomes or analyses was likely, including preferential reporting of significant results or the omission of relevant effect estimates (e.g., hazard ratios not reported despite Cox regression).	a. Low risk: All prespecified outcomes reported transparently.<br>b. Moderate risk: Some outcomes or analyses incompletely reported.<br>c. High risk: Clear evidence of selective reporting.

Table S4. Extended study characteristics

First_author_Year	Country	Study_design_(prospective/retrospective/other)	Inclusion_period	Sample_size_total	Sample_size_POLE_mut	Sample_size_POLE_wt_or_other	Age_mean_or_median_(years)	Stage_distribution_(FIGO)	Histology_distribution_(endometrioid/serous/other)	Grade_distribution_(1/2/3_or_low/high)	Molecular_subgroups_reported_(POLE/MMRd/p53abn/NSMP)	POLE_exonuclease_domain_regions_tested	Pathogenic_or_likely_pathogenic_POLE_definition_used	Sequencing_method_(NGS/Sanger/panel/other)	Cutoff_for_ultramutated_or_TMB_definition	Primary_outcome_(OS/DFS/PFS/CSS)	Reported_effect_measure_(HR/OR/RR)	HR_OS_(POLE_mut_vs_comparator)	95_CI_OS_lower	95_CI_OS_upper	HR_DFS_or_PFS	95_CI_DFS_or_PFS_lower	95_CI_DFS_or_PFS_upper	HR_CSS	95_CI_CSS_lower	95_CI_CSS_upper	Follow_up_median_(months)	Adjustment_variables_in_multivariable_models	Adjuvant_treatment_data_available_(Yes/No)	Adjuvant_treatment_summary_(chemo/radiotherapy/none)	Risk_of_bias_tool_used_(ROBINS-I)	Risk_of_bias_overall_judgment	Notes_(comments_on_variant_annotation etc.)
Aksahin E, 2025	Turkey	Retrospective cohort study	January 2000 – June 2022	114	8	106	61	I-II predominant	Endometrioid	I–III; POLE-mut more frequent in high-grade tumors (p=0.026)	POLE-mut 5–7%; MMR-d 43–47%; p53-mut ~5–10% ; NSMP ~42%	Exons 9–14	Pathogenic somatic mutations in POLE exonuclease domain, detected by Sanger sequencing; Novel exon 14 duplication (c.1368_1370dup, p.T457dup) reported	Sanger	NA	DFS and OS	Kaplan–Meier survival analysis; Cox proportional hazards regression (multivariate), but HRs for POLE vs comparator not explicitly reported	NA	NA	NA	NA	NA	NA	NA	NA	NA	65	Age, LVSI, grade, myometrial invasion, stage, POLE status, p53 status, MMR status, NSMP status	yes	Mixed adjuvant therapy; no significant difference between recurrence and control groups (details not stratified by POLE)	ROBINS-I	Moderate risk of bias: retrospective design, matched case–control subset single-center cohort	Survival advantage of POLE-mut is demonstrated descriptively; No extractable HR with 95% CI for POLE vs comparator is provided.
McConechy M, 2016	Canada	Retrospective cohort study	1983–2013	406	39	367	58 POLE-mut; 66 POLE wild-type	POLE-mut: 95% stage I; 2 cases stage II–III; 0 stage IV; POLE-wt: 68% stage I; 32% stage II–IV	POLE-mut: 82% endometrioid; Also serous, clear cell, mixed, undifferentiated present	POLE-mut: 49% grade 3; Grade 1–2 also present	Partial molecular classification reported	Exons 9–14	Somatic POLE exonuclease-domain mutations, hotspot (P286R/S, V411L) and non-hotspot variants; germline POLE excluded	Sanger	NA	PFS, DFS, OS, CSS	Hazard ratios (HR) with 95% CI, Cox proportional hazards models	0.69	0.22	1,67	0,48	0,1	1,48	0,22	0,02	0,83	62	Age, BMI, stage, grade, histology, LVSI, adjuvant treatment, POLE status	yes	Chemotherapy, pelvic EBRT, vaginal brachytherapy; interaction POLE × treatment not significant	ROBINS-I	Moderate risk of bias: retrospective design; long inclusion period; robust molecular testing and survival analysis mitigate bias	Landmark study validating prognostic impact of POLE EDM; eligible for quantitative meta-analysis
Van Gool I, 2018	Netherlands	Retrospective RCT	1980–1990	245	16	229	NA	100% Stage I	Endometrioid	NA	Only POLE-mutant vs POLE–wild-type analyzed	Exons 9, 13, and 14	Pathogenic somatic POLE proofreading-domain mutations; tumors with concurrent p53 mutation or MMR deficiency excluded	Sanger	NA	Recurrence-free survival	Hazard ratio (HR) with 95% CI, Cox regression with Firth correction (appropriate for zero-event group)	NA	NA	NA	0,143	0,001	0,996	NA	NA	NA	120	Adjustment limited due to zero events in POLE group; no multivariable HR table reported	yes	NA	ROBINS-I	strengths: randomized trial cohort, homogeneous stage; limitations: small POLE sample, single-arm analysis	Landmark clinical evidence that POLE prognostic benefit is independent of adjuvant therapy; Zero recurrences in POLE-mutant group
Stelloo E, 2016	Netherlands	Retrospective RCT	PORTEC-1: 1990–1997; PORTEC-2: 2000–2006	834	49	785	62 POLE-mutant; 68 overall cohort	I-II	Endometrioid	POLE-mutant: 26.6% grade 3; Majority grade 1–2	p53-mutant: 9%; MSI: 26%; POLE-mutant: 6%; NSMP: 59%	Exons 9 and 13	Hotspot pathogenic somatic POLE exonuclease-domain mutations	Sanger	NA	Recurrence-free survival; Distant recurrence; Disease-specific survival; Overall survival	Hazard ratios (HR) with 95% CI from Cox regression	0,907	0,367	2,237	0,869	0,116	6,532	NA	NA	NA	131	Age, grade, myometrial invasion, LVSI, adjuvant treatment, molecular subgroup	yes	NA	ROBINS-I	strengths: randomized trial population, central pathology review, very long follow-up; limitation: very small POLE subgroup (rare events)	One of the largest early-stage POLE cohorts; Confirms favorable prognosis descriptively; HRs are reported but statistically unstable due to near-zero events
Joe S, 2023	Korea	Rerospective cohort study	January 2013 – December 2018	183	29	154	55,9 overall	Stage I 73.8%, Stage II 26.2%	Endometrioid: 90.7%; Non-endometrioid: 9.3%	Low-grade: 79.8%; High-grade: 20.2%	POLEmut 15.9%; MMR-D 29.0%; p53abn 8.7%; NSMP 46.4%	Exons 9, 13, and 14	Pathogenic POLE EDM hotspot mutations (P286R, S297F, V411L, A456P, S459F)	PCR	NA	Recurrence-free survival (RFS); Overall survival (OS)	Cox proportional hazards regression	NA	NA	NA	NA	NA	NA	NA	NA	NA	73,5	Age, histology, grade, myometrial invasion, FIGO stage, prognostic risk group, molecular L1CAM classification	yes	None 80.3%; Radiotherapy 13.7%; Chemotherapy ± RT 6.0%	ROBINS-I	Moderate risk of bias (retrospective, single-center, low number of POLE events)	POLE prognosis demonstrated descriptively (0 recurrences, 0 deaths); ddPCR validated as a feasible alternative to NGS; Prognostic modeling focuses on combined molecular L1CAM classification, not POLE alone
Andrade DAP, 2024	Brazil	Prospective cohort	October 2020 – December 2022	114	18	96	63,1 overall, POLE-mute mean 57,7	IA 28 (24.6%), IB 28 (24.6%), II 8 (7.0%), IIIA 10 (8.8%), IIIB 4 (3.5%), IIIC 24 (21.0%), IVB 12 (10.5%)	Endometrioid 84 (73.7%); Serous 12 (10.5%); Clear cell 4 (3.5%); Carcinosarcoma 9 (7.9%); Mixed/Undifferentiated 5 (4.4%)	G1 5 (4.4%), G2 41 (36.0%), G3 68 (59.6%)	ProMisE: POLEmut 15.8%, MMRd 28.1%, p53abn 27.2%, NSMP 28.9%	Exon 9-14	POLE hotspot exonuclease domain” mutations (as per ProMisE methodology; hotspot sequencing)	Sanger	NA	OS and PFS	Kaplan–Meier + log-rank; Cox regression mentioned “if necessary” (numeric HRs not shown in pages available)	NA	NA	NA	NA	NA	NA	NA	NA	NA	23,2	NA	yes	None 16 (14.0%); Radiotherapy 45 (39.5%); Radiotherapy+chemotherapy 34 (29.8%); Chemotherapy 12 (10.5%); Unperformed (Stage IV) 7 (6.2%)	NA	NA	POLEmut: 0 recurrences (recurrence “Yes” = 0/18). At 24 months, POLEmut PFS 100% and OS 93.8% (KM summary table)
He D, 2020	China	Retrospective cohort study	2011-2016	426	38 (8,9%)	388	54	FIGO IA 72.5%; FIGO IB–IV 27.5%	Endometrioid 364; Serous 35; Clear cell 6; Mixed 11; Undifferentiated 6; Mixed serous/clear cell 4	Grade 1–2: 318 (74.6%); Grade 3: 108 (25.4%)	Partial (POLE mutation + MMR and p53 IHC status reported)	Exons 9, 13, and 14	Somatic missense mutations in POLE exonuclease domain (including P286R, V411L, Q453R; novel variants F274L, G420D, V460A)	Sanger	NA	OS and PFS	Kaplan–Meier + univariable Cox regression (HRs reported for	NA	NA	NA	NA	NA	NA	NA	NA	NA	55	Univariable Cox models; stratified analyses by POLE status including stage, myometrial invasion, MELF, LVSI, lymph node status	yes	Chemotherapy in 95 patients (22.3%); no radiotherapy administered	ROBINS-I	Moderate (retrospective, single-center)	POLE mutation associated with improved OS, but PFS not universally favorable, particularly in presence of MELF invasion. HRs are not

																	stratified analyses)																extractable for POLE vs comparator → narrative synthesis only; interaction effects (e.g., MELF × POLE) reported with large HRs.
Zong L, 2023	China	Retrospective cohort study	June 2010 – December 2018	335	42 (11,8%)	313	59	FIGO I 54.9%; II 6.6%; III 29.6%; IV 9.0%	Grade 3 endometrioid 177 (49.9%); Serous 48 (13.5%); Clear cell 39 (11.0%); Carcinosarcoma 19 (5.4%); UEC/DEC 6 (1.7%);	All tumors high-grade by definition (grade 3 EEC or non-EEC high-grade)	Yes (TCGA surrogate): POLEmut 11.8%, MMRd 29.9%, p53mut 36.1%, NSMP 22.2%	Exon 9-14	Validated pathogenic POLE exonuclease-domain mutations (P286R, V411L, S297F, A456P, S459F)	Sanger	NA	Relapse-free survival (RFS) and disease-specific survival (DSS)	Hazard ratios (HR) with 95% CI from univariable and multivariable Cox regression	NA	NA	NA	0	0	1,7	0	0	1,9	43	FIGO stage, LVSI status, molecular subtype	yes	Chemotherapy and/or radiotherapy according to ESGO risk groups (details stratified in text, not by POLE)	ROBINS-I	Moderate (retrospective, single-center, zero-event subgroup)	Largest single-institution cohort focused exclusively on high-grade EC. POLE-mut tumors showed no relapses or disease-specific deaths. HRs reported but not suitable for pooled meta-analysis due to zero events and extreme CI → include narrative / sensitivity only.
Leon-Castillo A, 2020	Multinational (Netherlands, UK, Australia, Canada, France)	Retrospective translational analysis of a randomized phase III trial	NA	410	51 (12,4%)	359	61,2 overall, 57,5 POLEmut	High-risk cohort; distribution reported overall: IA 13.2%; IB 17.8%; II 25.6%; IIIA 11.2%; IIIB 7.1%; IIIC 25.1%	Mixed high-risk population: endometrioid (grades 1–3), serous, clear cell, mixed, other; POLE-mut present across histologies	Endometrioid G1–2: 39.3%; G3: 27.6%; non-endometrioid: 28.6%	p53abn 22.7%; POLE-mut 12.4%; MMRd 33.4%; NSMP 31.5%	Exon 9-14	Pathogenic POLE exonuclease-domain mutations considered causative of ultramutated phenotype, per predefined criteria	NGS	NA	Recurrence-free survival (RFS) – primary; Overall survival (OS) – secondary	Hazard ratios (HR) with 95% CI from univariable and multivariable Cox regression	0,118	0,016	0,868	0,079	0,011	0,576	NA	NA	NA	73	Age, histology/grade, FIGO stage (I–II vs III), LVSI, adjuvant treatment, molecular subgroup	yes	Radiotherapy alone vs combined chemoradiotherapy (trial arms)	ROBINS-I	Low–moderate (randomized parent trial, robust molecular testing)	Landmark evidence that POLE-mut is the strongest favorable prognostic factor even in high-risk EC; excellent outcomes independent of adjuvant therapy. HRs stable and suitable for pooled analysis.
Han KH, 2024	Korea	Retrospective cohort study	January 2014 – December 2018	161	19 (11,8%)	142	57,2	FIGO 2009: I 55.9%; II 11.8%; III 26.7%; IV 5.6%; FIGO 2023 applied retrospectively (stages I–IV with molecular substaging)	Endometrioid 139 (86.3%); Serous 19 (11.8%); Clear cell 1 (0.6%); Carcinosarcoma 2 (1.2%)	Grade 1: 23.0%; Grade 2: 42.9%; Grade 3: 34.2%	Yes (TCGA surrogate): POLEmut 11.8%; MMRd 28.6%; p53abn 23.3%; NSMP 46.0%	Exons 9, 13, and 14	Pathogenic POLE exonuclease-domain hotspot mutations (P286R, S297F, V411L, A456P, S459F)	PCR	≥6 copies/20 µL or ≥0.3% mutation index	Progression-free survival (PFS), Overall survival (OS), Disease-specific survival (DSS)	Hazard ratios (HR) with 95% CI from multivariable Cox regression	NA	NA	NA	NA	NA	NA	NA	NA	NA	62,9	FIGO stage (2009/2023), molecular classification, histology, grade, LVSI, nodal status	yes	None 35.4%; Radiotherapy 24.2%; Chemotherapy 19.9%; Concurrent chemoradiotherapy 20.5%	ROBINS-I	Moderate (retrospective design, restaging study)	Study evaluates staging system performance, not POLE as an isolated prognostic factor. POLE-mut tumors show excellent PFS descriptively but no extractable POLE-specific HR → narrative synthesis only, exclude from pooled meta-analysis.
Leon-Castillo A, 2022	Danmark	Retrospective population-based cohort study	January 2005 – December 2012	367	38(10,4 %)	329	70 POLEmut	Stage I–III only (stage IV excluded): IA 46.9%; IB 27.0%; II 7.6%; IIIA 1.6%; IIIB 3.0%; IIIC 13.9%	Endometrioid G3 43.3%; Serous 34.1%; Clear cell 20.7%; Undifferentiated 1.9%	Exclusively high-grade tumors (grade 3 endometrioid and non-endometrioid)	yes	Exons 9, 13, and 14	Pathogenic POLE exonuclease-domain mutations defined per Leon-Castillo et al. criteria (ultramutated phenotype)	NGS	NA	Recurrence, Overall survival (OS), Disease-specific survival (DSS)	Hazard ratios (HR) with 95% CI from Cox regression	NA	NA	NA	NA	NA	NA	NA	NA	NA	97	Age, FIGO stage (I–II vs III), LVSI (absent/focal vs substantial), ASA score, adjuvant treatment, molecular subgroup; propensity score used to correct for treatment indication bias	yes	None 71.9%; Radiotherapy 9.3%; Chemotherapy 17.2%; Combined CRT 1.6%	ROBINS-I	Moderate (retrospective design, robust registry data, long follow-up)	Landmark evidence that POLE-mut high-grade EC has excellent prognosis independent of lymphadenectomy and adjuvant therapy. Zero-event issue precludes HR pooling; exclude from meta-analysis, include in qualitative synthesis.
Billingsley CC, 2016	United States	Retrospective cohort study	NA	72	7(9,7%)	65	64,6 overall; 58,3 POLE-mut	Stage I–II: 47 (65.3%); Stage III–IV: 24 (33.3%); Unstaged: 1 (1.3%)	Endometrioid	III	only POLE mut	Exonuclease domain residues 268–471	Somatic missense mutations in the POLE exonuclease (proofreading) domain	Sanger	NA	Recurrence-free survival (RFS) and overall survival (OS)	Adjusted hazard ratios (aHR) with 95% CI from Cox proportional hazards models (adjusted for age)	0,19	0,03	1,42	0,37	0,09	1,55	NA	NA	NA	38	Age only (models adjusted for age)	yes	Any adjuvant therapy in 41/72 (56.9%); specific modalities not stratified by POLE status	ROBINS-I	Moderate (small POLE subgroup, retrospective subanalysis)	POLE-mut tumors showed lower risk of recurrence and death, but estimates did not reach statistical significance due to small sample size and wide confidence intervals. HRs are reported and can be used in meta-analysis, with appropriate weighting and acknowledgment of imprecision.
Cosgrove CM, 2018	United States	Retrospective cohort study	2003–2007	982	39 (4%)	943	57,4	FIGO I 76.9%; II 0%; III 23.1%; IV 0% (POLE subgroup)	Endometrioid	Grade 1: 35.9%; Grade 2: 38.5%; Grade 3: 25.6% (POLE subgroup)	Copy number stable (CNS), MMR-deficient, copy number altered (CNA), POLE-mutant	Exons 9, 13, and 14	Pathogenic POLE exonuclease-domain mutations; POLE class assigned only if MMR-proficient and copy-number stable	Sanger	NA	Progression-free survival (PFS), Endometrial cancer–specific survival (ECS), Overall survival (OS)	Hazard ratios (HR) with 95% CI from Cox proportional hazards models	0,19	0,03	1,35	0,26	0,06	1,05	0,36	0,05	2,71	60,6	Age (>60), FIGO stage, grade, LVSI, adjuvant therapy, molecular group	yes	Any adjuvant therapy in 23.7% of POLE patients; majority managed with surgery alone	ROBINS-I	Moderate (observational prognostic analysis; excellent follow-up; rare-event subgroup)	Large multicenter EEC cohort demonstrating best survival for POLE-mut tumors, but HRs are imprecise due to rarity. Eligible for meta-analysis (HRs reported), with acknowledgment of wide CIs and rare-event bias.

Bosse T, 2018	Multicenter (Netherlands, Canada, USA, UK, Spain)	Retrospective cohort study	NA	381	49 (12,9%)	332	66 overall; 60 POLE mut	FIGO: IA 44.9%; IB 31.5%; II 6.3%; III 13.1%; IV 2.9%	Endometrioid	III	POLE 12.9%; MMRd 36.2%; p53abn 20.7%; NSMP 30.2%	Exon 9-14	Pathogenic POLE exonuclease-domain hotspot mutations, TCGA-ultramutated definition	Sanger	NA	Overall survival (OS) and Recurrence-free survival (RFS)	Hazard ratios (HR) with 95% CI from univariable and multivariable Cox regression	0,56	0,27	1,15	0,23	0,07	0,77	NA	NA	NA	73	Age (continuous), FIGO stage (IA / IB / II–IV), molecular subgroup	yes	Raeported globally	ROBINS-I	Moderate (retrospective, multicenter, robust follow-up)	Reference study for grade 3 endometrioid endometrial carcinoma. POLE-mutated tumors are associated with an excellent prognosis. Hazard ratios are clearly defined in comparison with the NSMP subgroup, not with "all other" cases.
Lindemann K, 2025	Norway	Retrospective cohort study	January 2006 – December 2017	360: Advanced EC (stage III/IV): 264; Recurrent EC: 96	Advanced cohort: 10 (4%); Recurrent cohort: 1 (1%)	Advanced cohort: 254; Recurrent cohort: 95	Advanced cohort: mean 67; Recurrent cohort: mean 68	Advanced cohort (FIGO 2009):IIIA 7%, IIIB 3%, IIIC1 33%, IIIC2 25%, IV 32%; Recurrent cohort: mixed stage I–IV at primary diagnosis	Mixed histology:Endometrioid (well/moderate/poorly differentiated); Serous carcinoma; Other histologies (30% advanced cohort)	NA	Advanced EC: POLE 4%, MMRd 25%, p53abn 44%, NSMP 26%; Recurrent EC: POLE 1%, MMRd 21%, p53abn 48%, NSMP 28%	Exon 9-14	Pathogenic POLE mutations according to ESMO / ProMisE criteria	Sanger	NA	Time-to-recurrence (TTR); Cancer-specific survival (CSS)	Hazard ratios (HR) with 95% CI from Cox regression models	NA	NA	NA	NA	NA	NA	0,32	0,08	1,32	60	FIGO stage, molecular subgroup, residual tumor status (stage-stratified analyses performed)	yes	Platinum-based chemotherapy (single, doublet, or triplet); minimal radiotherapy use	ROBINS-I	Moderate (retrospective, very small POLE subgroup)	POLE-mutated tumors show consistently favorable outcomes even in advanced/recurrent disease, but Event numbers are extremely low, making estimates unstable. Suitable for narrative synthesis and sensitivity analyses, but inclusion in pooled HR meta-analysis should be carefully justified.
Slomovitz BM, 2025	United States	Retrospective cohort study	January 2011 – September 2023	1139	12(1%)	1127	68 overall, 62 POLEmut	Stage I: 34.6%; Stage II: 5.5%; Stage III: 24.8%; Stage IV: 30.4%; Unknown: 4.7%	Endometrioid: 51.4%; Serous: 31.0%; Clear cell: 7.0%	NA	POLEmut: 1%; MSI-H: 22%; TP53mut: 47%; NSMP: 31%	Exons 9, 13, and 14	Known or likely pathogenic POLE exonuclease-domain alterations, classified according to TCGA/ProMisE criteria	Sanger	Ultramutated status inferred from POLE mutation; high TMB defined as ≥10 mutations/Mb (used descriptively)	Time to next treatment (TTNT); Overall survival (OS)	Hazard ratios (HR) with 95% confidence intervals from Cox proportional hazards models (univariable and multivariable)	0,66	0,21	2,08	NA	NA	NA	NA	NA	NA	60	ECOG performance status, stage at diagnosis, age, body mass index, self-identified race, molecular subgroup	yes	First-line treatment categories reported (chemotherapy alone, hormonal therapy, immune checkpoint inhibitor–based regimens, others); not stratified specifically by POLE status	ROBINS-I	Moderate (retrospective real-world design, extreme imbalance of molecular subgroups, very small POLE cohort)	This is a real-world, advanced/recurrent EC cohort. POLEmut tumors show a consistently favorable direction of effect, but event numbers are very small. HRs are validly reported vs NSMP, but precision is limited; inclusion in meta-analysis should be restricted to sensitivity or subgroup analyses.
Guo Q, 2024	China	Retrospective cohort study	2020–2022	331	47 (14.2%)	284	55	IA: 204 (61.6%); IB: 52 (15.7%); II: 20 (6.0%); III: 39 (11.8%); IV: 16 (4.8%)	Endometrioid: 284 (85.8%); Carcinosarcoma: 4.8%; Mixed carcinoma: 3.0%;	NA	POLE mut: 47 (14.2%); dMMR: 79 (23.9%); p53 abnormal: 57 (17.2%); NSMP: 148 (44.7%)	NA	Pathogenic/likely pathogenic POLE exonuclease-domain mutations defined according to WHO/TCGA molecular classification criteria	NGS	Ultramutated status inferred by presence of POLE exonuclease-domain mutation; explicit numeric TMB cutoff not applied	Disease-free survival (DFS)	Hazard ratios from Cox regression; Kaplan–Meier survival analysis	NA	NA	NA	NA	NA	NA	NA	NA	NA	32,6	Multivariable Cox regression performed; specific covariates not fully enumerated in main text	yes	Chemotherapy, radiotherapy, chemoradiotherapy, or no adjuvant therapy; POLE mut subtype showed excellent prognosis regardless of adjuvant treatment	ROBINS-I	Moderate (retrospective single-center design)	Large consecutive Chinese surgical cohort. Strong prognostic separation of POLE mut subtype demonstrated, but absence of HRs limits meta-analytic inclusion. Suitable for qualitative synthesis and subgroup context
Zammarrelli WA, 2022	United States	Retrospective cohort study	January 2014 – January 2020	75	24(32%)	51	61 overall, 56 POLEmut	IA: 56 (75%); IB: 19 (25%)	endometrioid	III	POLE: 24 (32%); MSI (MMRd): 26 (35%); Copy-number high (p53-abnormal): 15 (20%); Copy-number low (NSMP): 10 (13%)	Exon 9-14	Presence of POLE exonuclease-domain hotspot mutation identified by MSK-IMPACT sequencing	NGS	Ultramutated status inferred solely from POLE exonuclease-domain mutation; no	Progression-free survival (PFS); Overall survival (OS)	Hazard ratios (HR) with 95% confidence intervals from Cox regression models	0,23	0,05	1,53	0,08	0,01	0,7	NA	NA	NA	37	Molecular subtype, PORTEC-1 HIR, GOG-99 HIR, age, stage, depth of myometrial invasion, LVSI (as applicable)	yes	Radiotherapy: 92%; Chemotherapy: 28%	ROBINS-I	Moderate (retrospective, single-center, small event numbers)	Pure stage I grade 3 endometrioid cohort with centralized pathology review and comprehensive molecular classification. POLE-mutated tumors demonstrated excellent prognosis independent of adjuvant therapy. Suitable for PFS meta-analysis only if comparator is harmonized (e.g., POLE vs CN-H);

															numeric TMB cutoff applied																		otherwise narrative synthesis.
Kolehmainen A, 2020	Finland	Retrospective cohort study	January 2007 – December 2012	515	37 (7,2%)	478	66 overall; 59 POLEmut	IA: 75,7%; IB: 16,2%; II: 5,4%; III: 2,7%; IV: 0%	Endometrioid: 91.9%; clear cell 5,4%; seros: 2,7%	Endometrioid tumors only (n = 34 POLE):Grade 1: 61.8%; Grade 2: 23.5%; Grade 3: 14.7%	NSMP: 218; POLE: 37;MMR-D: 191; p53abn: 69	Exons 9, 13, and 14	Presence of POLE exonuclease-domain hotspot mutation (P286R, S297F, V411L, A456P)	Sanger	NA	Overall mortality, cancer-related mortality (primary), non-cancer-related mortality	Hazard ratios (HR) with 95% CI from univariable and multivariable Cox regression	NA	NA	NA	NA	NA	NA	NA	NA	NA	81	Age, FIGO stage, uterine risk factors, ER/PR expression, L1CAM expression, adjuvant therapy	yes	Vaginal brachytherapy, pelvic radiotherapy, chemotherapy, combined modalities; POLE subgroup mostly received none or radiotherapy only	ROBINS-I	Moderate (retrospective, single-center; robust follow-up)	Landmark clinical-molecular cohort using TransPORTEC classification. No cancer-related deaths in POLE subgroup, reinforcing favorable prognosis. Suitable for qualitative synthesis only; cannot contribute HRs to meta-analysis forest plots. POLE-mutant tumors serve as a prognostic gold standard for favorable outcomes.
Gonzalez-Bosquet J, 2022	United States	Retrospective cohort study	NA	192	28 (14.6%)	164	61	Stage I: 124 (75.6%); Stage II: 10 (6.1%); Stage III: 23 (14.0%); Stage IV: 7 (4.3%)	endometrioid	Grade 1: 38.4%; Grade 2: 39.6%; Grade 3: 22.0%	POLE ultramutated: 28; MSI-H (MMRd): 58; CNL / NSMP: 85; CNH / TP53-mutant: 21	Exons 9, 13, and 14	TCGA-defined ultramutated POLE exonuclease-domain mutations, used as a marker of favorable prognosis	WES	NA	Progression-free survival (PFS)	Hazard ratios (HR) with 95% CI from Cox proportional hazards models	NA	NA	NA	NA	NA	NA	NA	NA	NA	28,7	Age, grade, stage, depth of myometrial invasion, MSI-H, NSMP, TP53 mutation, HR gene mutations, CTNNB1, KRAS, ARID1A, PIK3CA, PTEN, PIK3R1, CIP2A expression, and ECPPF variables	yes	Predominantly platinum-based chemotherapy and/or radiotherapy in higher-risk cases; adjuvant therapy details not stratified by POLE status	ROBINS-I	Moderate (retrospective TCGA analysis; incomplete treatment annotation)	The study is highly relevant biologically, but not eligible for quantitative meta-analysis of POLE prognostic impact, as no HRs for POLE vs comparator are provided. Suitable for qualitative synthesis and contextual discussion only.

Table S5. Risk of Bias Ratings.

Study	D1. Bias due to confounding	D2. Bias in the selection of participants	D3. Bias in the classification of exposure (POLE status)	D4. Bias due to deviations from intended exposure	D5. Bias due to missing data	D6. Bias in the measurement of outcomes	D7. Bias in the selection of the reported result
**Aksahin E, 2025 [25]**	Moderate	Moderate	Low	Low	Moderate	Low	Moderate
**McConechy M, 2016 [26]**	Moderate	Moderate	Low	Low	Moderate	Low	Moderate
**Van Gool I, 2018 [27]**	Low	Moderate	Low	Low	Low	Low	Moderate
**Stelloo E, 2016 [28]**	Low	Low	Low	Low	Low	Low	Low
**Joe S, 2023 [29]**	Moderate	Moderate	Low	Low	Moderate	Low	Moderate
**Andrade DAP, 2024 [30]**	Moderate	Moderate	Low	Low	Moderate	Low	Moderate
**He D, 2020 [31]**	Moderate	Moderate	Moderate	Low	Moderate	Low	Moderate
**Zong L, 2023 [32]**	Moderate	Moderate	Low	Low	Moderate	Low	Moderate
**Leon-Castillo A, 2020 [5]**	Low	Low	Low	Low	Low	Low	Low
**Han KH, 2024 [33]**	Moderate	Moderate	Low	Low	Moderate	Low	Moderate
**Leon-Castillo A, 2022 [34]**	Low	Low	Low	Low	Low	Low	Low
**Billings-ley CC, 2016 [35]**	Moderate	Moderate	Low	Low	Moderate	Low	Moderate
**Cosgrove CM, 2018 [36]**	Moderate	Moderate	Low	Low	Moderate	Low	Moderate
**Bosse T, 2018 [37]**	Low	Low	Low	Low	Low	Low	Low
**Lindemann K, 2025 [38]**	Moderate	Moderate	Moderate	Low	Moderate	Low	Moderate
**Slomovitz BM, 2025 [39]**	Moderate	Moderate	Low	Low	Moderate	Low	Moderate
**Guo Q, 2024 [40]**	Moderate	Moderate	Low	Low	Moderate	Low	Moderate
**Zammarrelli WA, 2022 [41]**	Moderate	Moderate	Low	Low	Moderate	Low	Moderate
**Kolehmainen A, 2020 [42]**	Low	Low	Low	Low	Low	Low	Low
**Gonza-lez-Bos-quet J, 2022 [43]**	Moderate	Moderate	Low	Low	Moderate	Low	Moderate
